# Carriage of ESBL/AmpC-producing or ciprofloxacin non-susceptible *Escherichia coli* and *Klebsiella* spp. in healthy people in Norway

**DOI:** 10.1186/s13756-016-0156-x

**Published:** 2016-12-15

**Authors:** Charlotte R. Ulstad, Margrete Solheim, Sophie Berg, Morten Lindbæk, Ulf R. Dahle, Astrid L. Wester

**Affiliations:** 1 Domain for Infection Control and Environmental Health, Norwegian Institute of Public Health, Oslo, Norway; 2 Institute of Health and Society, University of Oslo, Oslo, Norway

**Keywords:** Faecal carriage, ESBL, Ciprofloxacin, Norway, *Escherichia coli*, *Klebsiella*

## Abstract

**Background:**

Asymptomatic carriage has been recognised as an important risk factor for infection caused by antibiotic resistant bacteria. A 14% global prevalence of Extended-Spectrum Beta-lactamase (ESBL) carriage was recently reported, but large intra-and interregional variations were observed. We investigated the faecal carriage rates of ESBL-, AmpC-producing and ciprofloxacin non-susceptible *Escherichia coli* and *Klebsiella* spp. in healthy Norwegians.

**Methods:**

Rectal samples were obtained from 284 volunteers, together with demographic data and information on recent travel history. The rectal samples were screened by selective plating and *E. coli* and *Klebsiella* spp. identified using MALDI-TOF. Phenotypic and molecular characterization of resistant isolates was also performed.

**Results:**

ESBL- or AmpC-producing *E. coli* and *Klebsiella* spp. were isolated from 4.9% and 3.2% of the study population, respectively. Carriage of ciprofloxacin non-susceptible isolates was detected in 9.9% of the volunteers. Molecular typing of ESBL/plasmid-mediated AmpC (pAmpC)-producing isolates suggested an allodemic situation rather than the dissemination of a specific clone in the Norwegian community. In concurrence with previous findings, travel to South-East Asia was associated with increased risk of carrying resistant *E. coli* or *Klebsiella* spp., highlighting the contribution of factors such as increased global mobility in erasing the boundaries between healthcare and community settings when it comes to spread of resistant bacteria.

**Conclusions:**

Overall, our study recognised Norway as a low-incidence country for faecal carriage of resistant bacteria among healthy individuals. Furthermore, our work denoted the importance of healthy humans as a reservoir for transmission of antibiotic resistant *E. coli* and *Klebsiella* spp.

**Electronic supplementary material:**

The online version of this article (doi:10.1186/s13756-016-0156-x) contains supplementary material, which is available to authorized users.

## Background

The prevalence of antimicrobial resistance (AMR) is increasing worldwide, and represents a serious threat to the global health [1, 2]. *Enterobacteriaceae* is one of the most common causes of both nosocomial and community acquired bacterial infections [3]. Traditionally, betalactam antibiotics and fluoroquinolones have been the treatment of choice for infections originating from Gram negative bacilli [2, 4]. However, the emergence of extended-spectrum betalactamases (ESBL and plasmid-mediated AmpC; pAmpC) and different mechanisms of ciprofloxacin resistance have rendered such infections notoriously challenging to treat and cure [4, 5].

Faecal carriage of ESBL probably represents the most important reservoir for infections with ESBL-producing *Enterobacteriaceae* [6, 7]. However, differences in the prevalence of gut colonization with ESBL-producing bacteria are observed both between and within regions, and the rates of colonization with ESBL-producing bacteria are generally increasing [8, 9]. Overall, an annual worldwide increase of 5.38% has been suggested [8]. CTX-M is the dominating ESBL-variant in communities worldwide [9]. Among clinical isolates found in Scandinavia, the predominant genotype is *bla*_CTX-M-15_ [10–12]. Data on community carriage of pAmpC is more limited, but it represents an important mechanism of resistance to extended-spectrum cephalosporins [13], although less common than ESBLs [14].

Reports on faecal colonization of ciprofloxacin-resistant *Enterobacteriaceae* are often based on the proportions of quinolone resistance in ESBL screening isolates, rather than screening for ciprofloxacin resistance in the first place. These observations may therefore be biased due to a significant association between ESBL production and ciprofloxacin resistance [15]. Prevalence studies in which ciprofloxacin resistance has been the primary criterion for selection are less frequent. The most recent data available corresponds to pre-travel colonization rates from studies reporting on travel-associated acquisition of resistant bacteria [16, 17].

Traditionally, Scandinavia is regarded as a low incidence area for antibiotic resistance [18]. Previous reports on faecal carriage in Sweden and Denmark confirm a favourable situation compared to most of Europe, including carriage among healthy volunteers [19–21]. In Norway, data on ESBL prevalence in clinical isolates is available through the Norwegian antibiotic resistance surveillance system (NORM). Two Norwegian studies report on faecal carriage rates of AMR bacteria. Rettedal et al. found that 2.9% and 0.3% of healthy pregnant women were colonised by ESBL-producing or AmpC-producing *E. coli*, respectively [22], whereas Jørgensen et al. observed an overall ESBL carriage rate of 15.8% in patients with diarrhoea, ranging from 10.3% in patients with no recent travel history to 56.3% in patients with a history of recent travel to Asia [23].

The primary objectives of this study were to determine the prevalence of ESBL/AmpC-producing and ciprofloxacin-resistant *E. coli* and *Klebsiella* spp. in healthy people in Norway. The data obtained may be used as an initial measurement in a time series evaluation of the prevalence of carriage among healthy humans in our country. In addition, we wanted to phenotypically characterise resistant isolates, and to determine the ESBL/pAmpC genotypes of the isolates identified.

## Methods

### Participants and collection of faecal samples

Healthy Norwegians volunteered to participate in the study from October 2014 to March 2016. They were recruited by general practitioners located in different parts of Norway, at health-related universities and other health institutions. Exclusion criteria were as follows: 1) recent acute gastroenteritis, 2) chronical illness which implies immunosuppression, 3) repeated hospitalisations, and 4) use of antibiotics within the past year. In a written questionnaire, each participant provided information on age, gender, county of residence, and travel abroad during the past 3 and 12 months. They also provided a faecal sample from their rectum using FecalSwab™ (Copan Italy, Brescia, Italy), and delivered it by mail together with the questionnaire to the National reference laboratory of enteropathogenic bacteria at the Norwegian Institute of Public Health (NIPH). Samples and questionnaires were identified by study-ID numbers only. The samples were analysed upon arrival, or stored at -70 degrees until analysed. All participants provided informed consent.

### Isolation of resistant *E.coli *and *Klebsiella* spp.

From each participant’s sample, the rectal swab was removed and 100 μl of Cary-Blair medium were spread onto MacConkey agar plates, supplemented with cefotaxime (1 mg/L; Duchefa Biochemie, Haarlem, the Netherlands), ceftazidime (2 mg/L; Sigma Aldrich, St. Louis, US), ciprofloxacin (0,125 and 0,25 mg/L; Fluka Chemicals, Buchs, Switzerland), and one control plate without supplementation. In addition, 200 μl and 400 μl of Cary-Blair medium were added into two separate tubes with MacConkey broth supplemented with 1 mg/L cefotaxime. Agar plates and broths were incubated overnight at 35 °C. The following day, the broths were spread to MacConkey agar plates with cefotaxime (1 mg/L), and incubated overnight at 35 °C. Single colonies of *E.coli* or *Klebsiella* spp. were selected from the different plates. If multiple morphologies were observed, all unique morphotypes were selected. Species identification was performed using MALDI-TOF MS (Bruker Daltonik GmbH, Bremen, Germany). Samples that yielded no, or sparse growth on the MacConkey control plate, were excluded from the study.

### Antibiotic susceptibility testing and ESBL identification

Antibiotic susceptibility testing (AST) against ciprofloxacin was performed using MIC (minimal inhibitory concentration) strip test (Liofilchem, Abruzzi, Italy), according to EUCAST guidelines and interpreted according to NORDICAST Clinical Breakpoints [24]. AST against a broad range of other antibiotics (ampicillin, amoxicillin-clavulanic acid, azetronam, cefotaxime, cefoxitin, cefuroxime, ceftazidime, gentamicin, imipenem, meropenem, mecillinam, nalidixic acid, piperacillin-tazobactam, and temocillin) was performed using the disc diffusion (BD Sensi-Disc, Becton-Dickinson, Sparks, USA) according to EUCAST guidelines (EUCAST disk diffusion method, v. 5.0, January 2015), and interpreted according to NORDICAST Clinical Breakpoints (or EUCAST epidemiological cut-offs (ECOFFs), if clinical breakpoint were not available). For meropenem, isolates with a zone diameter narrower than the NORDICAST screening breakpoint (<27 mm) were submitted to the Norwegian National Advisory Unit on Detection of Antimicrobial Resistance (K-res) for further characterization. Phenotypic confirmation of ESBL or AmpC was performed using the Total ESBL + AmpC Confirm kit (Rosco Diagnostica, Denmark). This kit utilises tablets containing cefotaxime or ceftazidime in combination with β-lactamase inhibitors (i.e. clavulanate and/or cloxacillin) to detect ESBL and AmpC production phenotypically. Results were interpreted according to manufacturer's instructions, by comparing the inhibition zones of the different tablets and thereby identifying synergy effects. *E.coli* ATCC25922 and two strains of *K. pneumoniae* (CTX-M 15) and *Providencia stuartii* (CMY-2), obtained from K-res, were used as controls. Isolates either resistant or intermediately sensitive to ciprofloxacin were categorised as ciprofloxacin non-susceptible, whereas isolates non-susceptible to three or more groups of antibiotics were categorised as multi-drug resistant (MDR; [25]).

### Differentiation of multiple isolates obtained from the same participants

An in house MLVA scheme was used together with phenotypic resistance profiles to differentiate multiple ESBL/AmpC-producing isolates from the same faecal sample. Bacterial DNA was prepared by boiling lysis (100 °C for 15 min, followed by 3 min. centrifugation at 14000 × g). MLVA was performed by targeting 10 tandem repeats (CVN001, CVN002, CVN003, CVN004, CVN007, CVN014, CVN015, CCR002, CVN016, CVN017), as previously described [26].

### Molecular characterization of ESBL and pAmpC types

*E. coli* and *Klebsiella* spp. displaying an ESBL or AmpC phenotype were screened for ESBL-and pAmpC encoding genes by multiplex PCR. The primers used for PCR are listed in Additional file 1: Table S1. PCR was carried out as previously described [26]. PCR products were separated and visualised using the Bioanalyzer DNA 1000 system (Agilent Technologies, Santa Clara, US) according to the manufacturer’s protocol.

Isolates with positive PCR results were selected for whole-genome sequencing (WGS) to further characterise the mechanism of resistance. Cells were grown over night in Luria Broth (Sigma Aldrich, St. Louis, US) with shaking at 35 °C, and DNA was extracted from 1 ml culture using the Wizard Genomic DNA Kit (Promega, Madison, US) according to the manufacturer's instructions. Quantification of total DNA was performed using a Qubit fluorometer (Life Technologies, Waltham, US), with broad range or high specificity reagents as appropriate. The sequencing libraries were prepared with the Nextera XT DNA Sample Prep Kit (Illumina, Eindhoven, the Netherlands) and sequencing was performed using a MiSeq sequencer (Illumina) in a 2 × 150-bp paired-end run. AMR genes were identified from WGS data using ResFinder [27] and Arg-annot [28].

### Statistical analysis

Statistical analyses were performed using SPSS (SPSS Inc., Chicago, Illinois). Chi squared test and Fisher’s exact test were used, as appropriate. A p-value of <0.05 was considered statistically significant. Odds Ratios (OR) with 95% confidence intervals (95% CI) were computed manually. For calculations of OR, the group “Not travelled/Travelled within Scandinavia” was treated as a reference.

## Results

### Participants

**Fig. 1:**
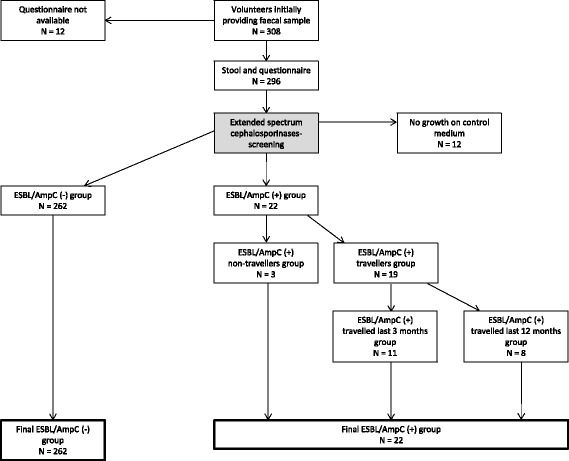
Study protocol for ESBL- and AmpC prevalence. Study protocol for investigating prevalence of extended-spectrum beta-lactamase (ESBL and AmpC)-producing *E.coli* and *K. pneumoniae* among healthy Norwegians, in travellers and non-travellers. One volunteer was carrier of both ESBL- and AmpC-producing isolates

Rectal samples were obtained from 308 healthy individuals, of whom 296/308 (96.1%) returned the questionnaire (Fig. 1). Samples from an additional 12/308 (3.9%) volunteers showed no growth on control media, and were therefore excluded from the study, leaving the remaining 284/308 (92.8%) eligible for further analyses. 174/284 (61.3%) were female and 102/284 (35.9%) were male (Table 2). Eight participants did not provide gender information. Fifty-three subjects (18.7%) belonged to the age category 18-29 years, whereas 108 (38.0%), 76 (26.8%) and 44 (15.5%) belonged to the age categories 30-49 years 50-64 years 65-84 years, respectively (Table 2). Three subjects (1.0%) did not provide information about age. The volunteers resided in fourteen of 19 different Norwegian counties (results not shown).

### Prevalence and characterization of ESBL

ESBL-producing strains were isolated from 14/284 healthy volunteers (4.9%). From the fourteen positive samples, 16 different ESBL-producing isolate were obtained, *i.e.* two individuals carried more than one ESBL-producing isolate. Overall, 15/16 isolates were identified as *E.coli* (93.8%) and 1/16 (6.2%) as *K. pneumoniae.* One of the two carriers of more than one isolate was colonised with both *E.coli* and *K. pneumoniae.*

The β-lactamase genes of all ESBL-producing isolates were further characterised by PCR and sequencing. For one ESBL-producing isolate, SHV was detected by PCR, but WGS yielded no known ESBL-gene, and this isolate will therefore be further investigated. Among the remaining isolates, CTX-M accounted for 13/16 (86.7%) of the ESBL production, with CTX-M1 and CTX-M9 as the dominant CTX-M groups (Table 1). *bla*_CTX-M-15_ was the dominant genotype (6/16; 37.5%). One isolate showed co-occurrence of *bla*_CTX-M-1_ and *bla*_TEM-210_, and in an additional 4/16 (25%) isolates a variant of *bla*_CTX-M_ was detected in combination with *bla*_TEM-1B._ The *E.*coli and *K. pneumoniae* isolates originating from the same volunteer harboured *bla*_CTX-M-15_ and *bla*_SHV-12_, respectively.

**Table 1:** The dominating ESBL-genotypes isolated from *E.coli* and *Klebsiella pneumoniae* from healthy people in Norway. For one of the isolates obtained, the genotype could not be determined

ESBL family	ESBL gene identified	Number of isolates (% of total)
CTX-M-1	*bla*_CTX-M-15_	6 (40)
	*bla*_CTX-M1_ + *bla*_TEM-210_	1 (6.7)
	*bla*_CTX-M-55_	1(6.7)
CTX-M-9	*bla*_CTX-M-3_	2 (13.3)
	*bla*_CTX-M-27_	2 (13.3)
	*bla*_CTX-M-14_	1 (6.7)
	*bla*_CTX-M-24_	1 (6.7)
Other	*bla*_SHV-12_	1 (6.7)
Total		15

**Fig. 2:**
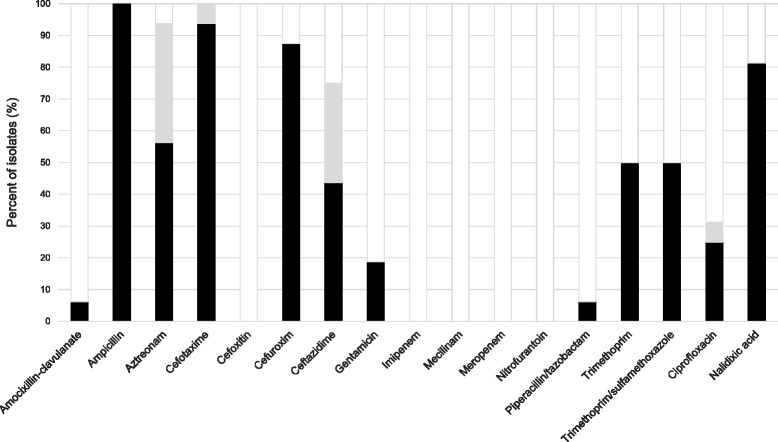
Antibiotic susceptibility among ESBL-producing isolates. Antibiotic susceptibility of faecal ESBL-producing *E.coli* and *K. pneumoniae* isolated from healthy Norwegians. Resistant (*black*); intermediate (*grey*), susceptible (*white*)

**Fig. 3:**
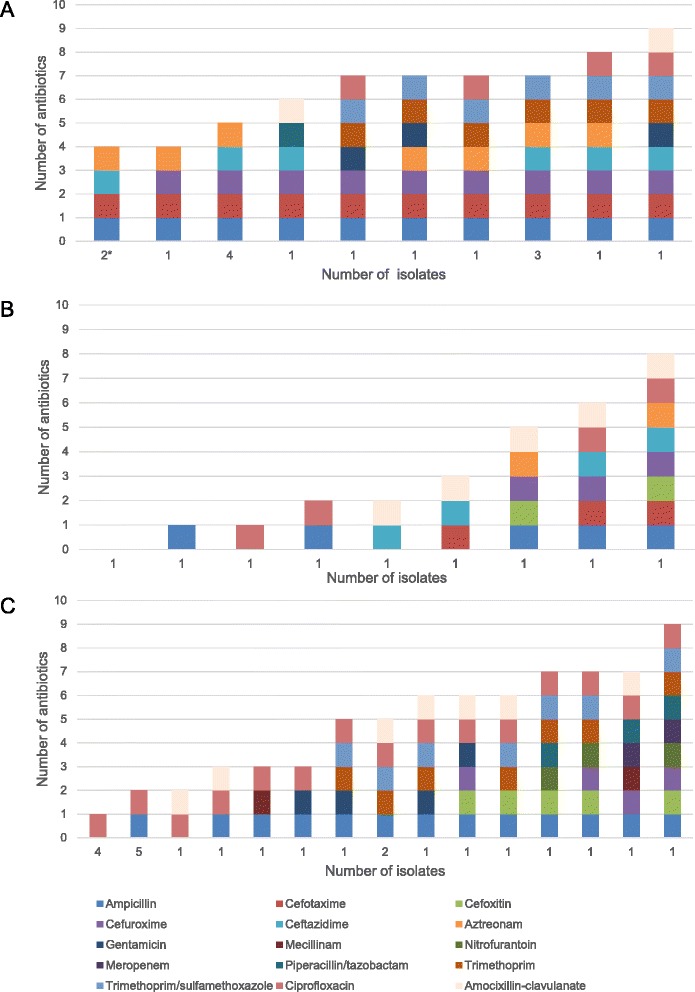
Antibiotic resistance profiles for resistant isolates. The number of isolates that displayed each unique resistance profile among the **a**) ESBL-producing, **b**) AmpC-producing, and **c**) ciprofloxacin non-susceptible *E.coli* and *K. pneumoniae* isolates obtained from healthy Norwegians. The numbers on the x-axis denote the number of isolates with that exact resistance profile. Both resistant and intermediate phenotypes are included. *Klebsiella* spp. are intrinsically resistant to ampicillin, and profiles recovered from isolates identified as *Klebsiella* spp. are marked with an asterisk (*)

ESBL-producing *E. coli* and *K. pneumonia* isolates displayed resistance to multiple other classes of antimicrobial agents as well (Fig. 2 and Additional file 1: Table S2), and all of them (16/16; 100%) were by definition MDR. One isolate displayed resistance against 8 antimicrobial drug groups, and 10 antimicrobial agents (Fig. 3a).

### Prevalence and characterisation of pAmpC

Furthermore, 9/284 (3.2%) of the volunteers were colonised by AmpC-producing *E.coli*. The methodology used for phenotypic characterisation does not distinguish between chromosomally encoded and pAmpC resistance and only 2/9 of the isolates were confirmed as pAmpC by PCR and WGS. Isolates from which negative PCRs were obtained were considered hyperproducers of chromosomally encoded AmpC. The two pAmpC-producing isolates were obtained from two different individuals, resulting in a carriage rate of 0.7% (2/284) for pAmpC.

**Fig. 4:**
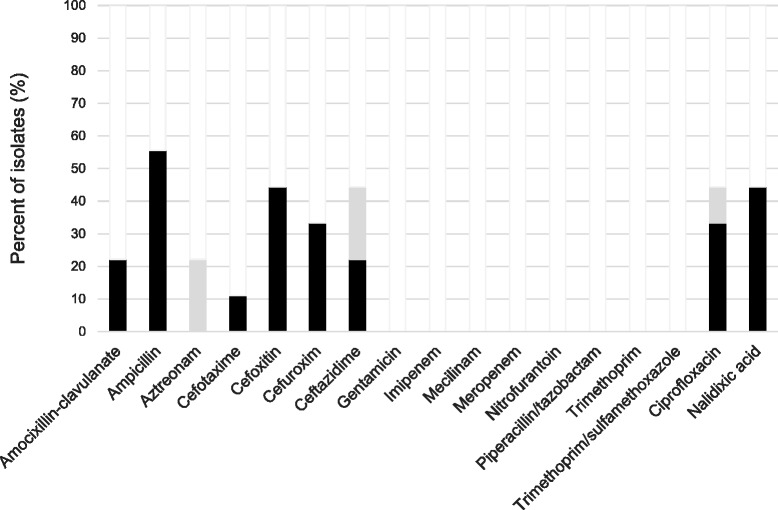
Antibiotic susceptibility among AmpC-producing isolates. Antibiotic susceptibility of faecal AmpC-positive *E.coli* isolated from healthy Norwegians. Resistant (*black*); intermediate (*grey*), susceptible (*white*)

Of the two pAmpC-producing isolates, one was found to harbour *bla*_DHA-1_ and the other *bla*_CMY-2_ in combination with *bla*_TEM1-C_ (Table 1). No co-producers of ESBL and pAmpC were recovered; however, one volunteer carried both an ESBL (*bla*_CTX-M-15_) - and a pAmpC-producing isolate (*bla*_DHA-1_). The nine AmpC-producing isolates displayed resistance to additional classes of antibiotics (Fig. 4 and Additional file 1: Table S3). 4/9 (44.4%) of the isolates were MDR (Fig. 3b). The isolates that harboured *bla*_DHA-1_ and *bla*_CMY-2+TEM-1C_ were resistant to 5 and 7 antimicrobial drug groups, respectively.

### Prevalence and characterization of isolates non-susceptible to ciprofloxacin

**Fig. 5:**
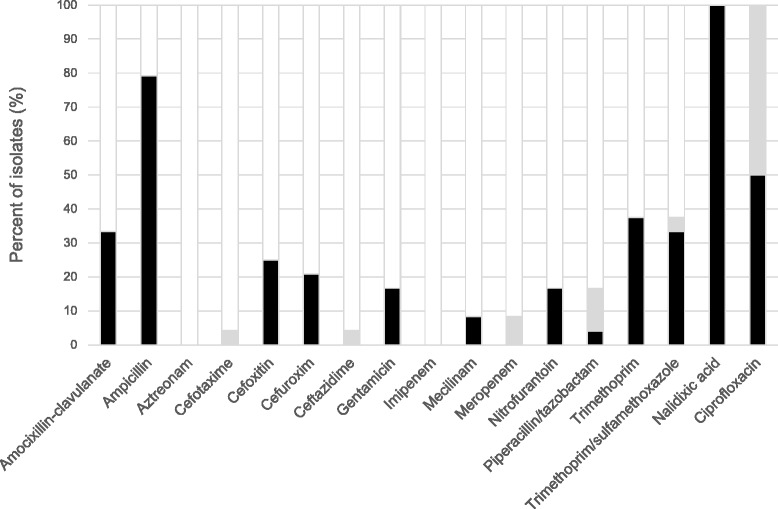
Antibiotic susceptibility among ciprofloxacin non-susceptible isolates. Antibiotic susceptibility of faecal ciprofloxacin non-susceptible *E.coli* and *K. pneumoniae* isolated from healthy Norwegians. Resistant (*black*); intermediate (*grey*), susceptible (*white*)

A total of 28/284 (9.9%) volunteers carried *E.coli* or *Klebsiella* spp. isolates that had MIC-values indicating non-susceptibility to ciprofloxacin; of which half (14/28) carried isolates displaying intermediate resistance to ciprofloxacin (MIC 0.5-1 mg/L; Fig. 5 and Additional file 1: Table S4). From the 28 subjects, 33 isolates were recovered; of which 5/33 (15.2%) were ESBL positive and 4/33 (12.1%) were AmpC positive. Of the remaining, 4/24 (16.7%) were *K. pneumoniae* and 20/24 (83.3%) were *E.coli*. The susceptibility of the ciprofloxacin non-susceptible isolates to several other classes of antibiotics was also tested (Fig. 5 and Additional file 1: Table S4) and 12/24 (50.0%) were categorised as MDR, including all the *K. pneumoniae* isolates (Fig. 3c). Meropenem zone diameters just below the NORDICAST screening breakpoint were observed for two non-ESBL/AmpC-producing ciprofloxacin non-susceptible isolates (Fig. 3c, 5 and Additional file 1: Table S4), and these isolates were thus submitted to K-res for further characterisation. However, none of the isolates were found to be carbapenemase-producing.

The ECOFF values for ciprofloxacin are set considerably lower than the clinical breakpoint for both *E.coli* and *Klebsiella* spp. If ECOFFs had been applied to categorise susceptibility in the present study, 66 ciprofloxacin non-susceptible isolates were obtained from 56/284 (19.7%) volunteers.

### Risk factors

The associations between faecal carriage of ESBL/AmpC-producing- and/or ciprofloxacin non-susceptible *E. coli* and *Klebsiella* spp. and various individual factors were assessed (Table 2). There were no significant associations between colonisation with resistant bacteria, and gender, age or county of resident (not shown) among the healthy volunteers.

**Table 2:** Characteristics of the participants and associations with faecal carriage of resistant bacteria

Characteristics	Participants (% of total)	Isolates with ESBL/AmpC production alone			Isolates with ciprofloxacin-non susceptibility alone			Isolates with both ESBL/AmpC *and* ciprofloxacin non-susceptibility		
		EAP (% within group)	Non-EAP (% within group)	*p*-value	CNS (% within group)	Non-CNS (% within group)	*p*-value	EAP + CNS (% within group)	Non EAP + CNS (% within group)	*p*-value
**Gender** (information missing for 8)										
Female	174 (61.3)	11 (6.3)	163 (93.6)	.746	11 (6.3)	163 (93.6)	.375	5 (2.9)	169 (97.1)	1.0
Male	102 (35.9)	4 (3.9)	98 (96.1)		11 (10.8)	91 (89.2)		3 (2.9)	99 (97.1)	
**Age group** (years; information missing for 3)										
18-29	53 (18.7)	6 (2.1)	47 (97.9)	.222	3 (5.7)	50 (94.3)	.944	1 (1.9)	52 (98.1)	.239
30-49	108 (38.0)	4 (3.7)	104 (96.3)		8 (7.4)	100 (92.6)		1 (0.9)	107 (99.1)	
50-64	76 (26.8)	4 (5.3)	72 (94.7)		6 (7.9)	70 (92.1)		4 (5.3)	72 (94.7)	
65-84	44 (15.5)	1 (2.3)	43 (97.7)		4 (9.1)	40 (90.9)		2 (4.5)	42 (95.5)	
**Geographic regions# visited last 3 months**										
Not travelled /travelled within Scandinavia	194 (68.3)	9 (4.6)	185 (95.4)		11 (5.7)	183 (94.3)		4 (2.1)	190 (97.9)	
Europe (outside Scandinavia)	69 (24.3)	2 (2.9)	67 (97.1)	**.002**	6 (8.7)	63 (91.3)	**.040**	2 (2.9)	67 (97.1)	.145
America	1 (0.4)	0 (0)	1 (100)		0 (0)	1 (100)		0 (0)	1 (100)	
Eastern Mediterranean	2 (0.7)	1 (50)	1 (50)		0 (0)	2 (100)		0 (0)	2 (100)	
South-East Asia	4 (1.4)	3 (75)**	1 (25)		1 (25)	3 (75)		1 (25)	3 (75)	
Western Pacific	2 (0.7)	0 (0)	2 (100)		1 (50)	1 (50)		0 (0)	2 (100)	
Africa	1 (0.4)	0 (0)	1 (100)		0 (0)	1 (100)		0 (0)	1 (100)	
Multiple regions	11 (3.9)	0 (0)	11 (100)		3 (27.3)*	8 (72.7)		1 (0.9)	10 (99.1)	
**Geographic regions# visited last 12 months**										
Not travelled /travelled within Scandinavia	101 (35.6)	5 (5.0)	96 (95.0)		8 (7.9)	93 (92.1)		1 (0.6)	100 (99.4)	
Europe (outside Scandinavia)	133 (46.8)	6 (4.5)	127 (95.5)	.089	6 (4.5)	127 (95.5)	.051	3 (2.3)	130 (97.7)	**.045**
America	1 (0.4)	0 (0)	1 (100)		0 (0)	1 (100)		0 (0)	1 (100)	
Eastern Mediterranean	2 (0.7)	1 (50)	1 (50)		0 (0)	2 (100)		0 (0)	2 (100)	
South-East Asia	2 (0.7)	1 (50)	1 (50)		0 (0)	2 (100)		1 (50)*	1 (50)	
Western Pacific	1 (0.4)	0 (0)	1 (100)		1 (100)	0 (0)		0 (0)	1 (100)	
Africa	1 (0.4)	0 (0)	1 (100)		0 (0)	1 (100)		0 (0)	1 (100)	
Multiple regions	43(15.1)	2 (4.7)	41(95.3)		7 (16.3)	36 (83.7)		3 (7.0)	40 (93.0)	
Total	284 (100)	15 (5.3)	269 (94.7)		22 (7.7)	262 (92.3)		8 (2.8)	276 (97.2)	

EAP = ESBL-or AmpC-producing, CNS = ciprofloxacin non-susceptible. Significant *p*-values are given in bold. Carriers of multiple isolates are represented one time per isolate *if* the isolates belong to different resistance groups, and percentage in the table may thus deviate from percentage presented in the text. *E.g.* both ciprofloxacin non-susceptible isolates and AmpC-producing ciprofloxacin non-susceptible isolates were recovered from two subjects, whereas both an ESBL-producing isolate and an AmpC-producing ciprofloxacin non-susceptible isolate were obtained from a third subject. Consequently, the first two subjects are represented both in the CNS and the EAP + CNS columns, while the third subject is represented both in the EAP and the EAP + CNS columns

#WHO regions

*Significantly different from the Not travelled/travelled within Scandinavia group, which was treated as a reference (*p* < 0.05)

**Significantly different from the Not traveled/travelled within Scandinavia group, which was treated as a reference (*p* < 0.005)

Univariate analysis of travel information recognised travel to South-East Asia during the last 3 months as a risk factor of faecal carriage of ESBL/AmpC-producing *E. coli* or *Klebsiella* spp. (OR 61.67; 95% CI 5.82-653.13), whereas travel to South-East Asia during the last 12 months was associated with increased risk of faecal carriage of ESBL/AmpC-producing ciprofloxacin non-susceptible *E. coli* or *Klebsiella* spp. (OR 100; 95% CI 3.34 to 2997.88). ESBL/AmpC-producing isolates were recovered from 75% (3/4) of travellers to South-East Asia compared to 3.9% (11/280) for those with no recent travel history or those who had travelled to other regions during the last 3 months (Table 2). Of note, all three carriers of more than one ESBL/AmpC-producing isolate had been visiting South-East Asia. Furthermore, travel to multiple WHO regions during the last 3 months (OR 6.24; 95% CI 1.45-26.86) was significantly associated with faecal carriage of ciprofloxacin non-susceptible *E. coli* or *Klebsiella* spp. The prevalence of ciprofloxacin non-susceptible isolates among travellers to multiple WHO regions (Additional file 1: Figure S2) during last 3 months was 27.3% (3/11) compared to 7.0% (19/273) for those who did not travel or travelled to one region only (Table 2).

## Discussion

The present study was undertaken to assess community carriage rates of antibiotic resistant *E. coli* and *Klebsiella* spp. in Norway. From 284 volunteers, we found that 4.9% were colonised with ESBL- and 3.2% with AmpC-producing *E. coli* or *Klebsiella* spp. Of the latter, the proportion of plasmid-mediated resistance corresponded to a carriage rate of pAmpC-producing *E. coli* or *Klebsiella* spp. of 0.7%. Our results were thus consistent with an ESBL colonisation rate of 3-6% in Europe [8]. In Scandinavia, the ESBL carriage rate has traditionally been lower than that in other parts of Europe; however, numbers are rising here as well. A recent report from Sweden documented that the faecal carriage among elderly subjects varied from 8.7% to 11%, depending on the living situation [29]. Our findings thus indicate a lower carriage rate among healthy individuals in Norway than in Sweden. Still, an increase in cephalosporin resistance rates has been observed among clinical isolates in Norway from the turn of the century [10], and it is likely that the colonisation level of healthy individuals in the country is following the same trend.

In accordance with epidemiology worldwide [8], the majority of ESBL-positive isolates in the present study were *E.coli* and the predominant ESBL allele was *bla*_CTX-M-15_. However, the diversity of genotypes detected suggests simultaneous community spread of various ESBL genotypes, as opposed to spread of this particular ESBL gene. An overlap in the distribution of genotypes between community and clinical settings is observed for both ESBL and pAmpC, indicating that resistant microbial populations are shared between hospitals and community [10]. However, the ratio between ESBL/AmpC-producing *Klebsiella* spp. and *E.coli* is considerably lower than the ratio observed in healthcare settings [10], indicating a difference in transmission dynamics between *E. coli* and *Klebsiella* spp. This is in line with previous findings, and suggests that ESBL/AmpC-producing *E.coli* is more likely to spread in the community [30, 31].

Ciprofloxacin non-susceptible isolates were recovered from 28/284 (9.3%) volunteers. A positive correlation has been reported between total usage of fluoroquinolones and the prevalence of fluoroquinolone non-susceptibility among clinical isolates in Norway [10], mirroring the internationally observed situation [32]. There is a notable lack of recent data on community carriage of ciprofloxacin resistant isolates in Europe, and our study thus adds new and significant information on the situation. Increased knowledge on prevalence and trends in resistance development can, together with information on antibiotic use, assist in the evaluation of any measures taken to control antibiotic resistance.

NORDICAST clinical breakpoints rather than ECOFFs were primarily applied to categorise susceptibility herein. For ciprofloxacin, the application of ECOFFs, in addition to clinical breakpoints, enabled us to differentiates between ‘percentage clinical resistant’ and ‘percentage decreased susceptible’ isolates. Indeed, considerable differences were observed between the two populations: ~15% of participants can be suspected to carry isolates with an acquired or mutational mechanism of ciprofloxacin resistance of unknown clinical relevance. Decreased susceptibility to fluoroquinolones is associated with decreased clinical responses to fluoroquinolones in *Salmonella* infections [33]. The marked differences in prevalence obtained by the application of clinical breakpoints compared to ECOFF values demonstrate the relevance of applying both cut-offs in studies like the present, as application of only clinical breakpoints can mask important shifts in MICs.

In our study, we found that all ESBL/pAmpC-producing and > 50% of the ciprofloxacin non-susceptible isolates, including all ciprofloxacin non-susceptible *Klebsiella* isolates were MDR. High rates of community faecal carriage of MDR isolates contribute to an increase in colonisation pressure and highlight the need for appropriate infection control policies.

Several reports identify travel as a risk factor of acquiring EBSL-producing isolates, with India and South-East Asia as high risk travel destinations [8, 34–36]. This is in agreement with our findings. A Dutch study found that travel to Asia is also a risk factor of being colonised with ciprofloxacin-resistant isolates [17]. The data presented herein recognise travel to multiple WHO regions within the same time frame, as a risk factor for being colonised with ciprofloxacin non-susceptible *E. coli* and *Klebsiella* spp. The majority of the visitors to multiple WHO regions reported South-East Asia or Western Pacific as one of the regions visited (Additional file 1: Figure S1). This is in line with the findings of Reuland et al. [17].

A potential limitation of our study was that the participants were not representative for the Norwegian population according to gender and county of residence. Most of the participants were female and live in the eastern part of Norway. However, only minor geographical differences in the prevalence of ESBL have been observed among clinical isolates in Norway [10], and it is likely that this observation can be extrapolated into community settings as well. Moreover, many of the volunteers were recruited via general practitioners and medical teaching institutions, where it is possible that augmented exposure to resistant bacteria can contribute to an overestimation of prevalence. Rigid exclusion criteria were therefore applied to reduce biases related to skewed individual recruitments. The employment of stringent exclusion criteria confounds recruitment of participants to the study, but adds validity to the associated findings. However, conclusions based on results from regions with small numbers of travellers should be made with caution. Furthermore, the sensitivity may have been decreased, because of insufficient self-sampling, storage conditions and by the sending of samples by regular mail. However, a sampling kit optimised for transport and preservation of faecal samples were chosen to minimise this effect.

The frequency of AMR in clinical isolates in Norway is well-documented through NORM, and although increasing, it continues to be low when compared to other parts of Europe. An ambitious national strategy against antibiotic resistance, together with the low prevalence of antibiotic resistance in Norway, offers a unique opportunity to gain knowledge on how to effectively prevent faecal colonisation with resistant *Enterobacteriaceae* in the community. As stated in the WHO Global action plan on AMR [37], surveillance is one of the main strategic objectives for preventing further spread and development of AMR worldwide. In order to strengthen our knowledge base, it is pivotal to monitor AMR trends consistently over time. Community carriage rates constitute an important source for information regarding the AMR situation in different populations, and AMR surveillance systems should thus be expanded to cover community carriers as well, *e.g.* by implementing a sampling campaign as part of the European Antibiotic Awareness Day.

## Conclusions

In conclusion, our study recognises Norway as a country with low prevalence of AMR carriage in the intestinal flora in healthy individuals. ESBL- producers were obtained from 4.9% of the study population, whereas AmpC-producers were obtained from 3.2%. Of the latter, the proportion of pAmpC corresponded to an overall carriage rate of 0.7%. In comparison, the carriage rate of ciprofloxacin non-susceptible isolates was 9.9%. A high proportion of intermediately ciprofloxacin resistant isolates may represent a shift in the ciprofloxacin MIC away from fully susceptible wild-type populations. Overall, our study denotes the importance of healthy humans as a reservoir for transmission of antibiotic resistant *E. coli* and *Klebsiella* spp., even in low incidence countries.

## Additional file

Additional file 1: Supplemental material. (DOCX 32 kb)

## Abbreviations

AMR: antimicrobial resistance
AST: antibiotic susceptibility testing
ECOFF: epidemiological cut-off
ESBL: extended-spectrum betalactamases
EUCAST: European committee on antimicrobial susceptibility testing
K-RES: Norwegian National Advisory Unit on Detection of Antimicrobial Resistance
MDR: multidrug resistant
MIC: minimal inhibitory concentration
NIPH: Norwegian Institute of Public Health
NORM: Norwegian surveillance system for antimicrobial resistance (Norsk overvåkingssystem for antibiotikaresistens hos mikrober)
UTI: urinary tract infection
WHO: World health organization
