# Supplementary material for: Carriage of ESBL/AmpC-producing or ciprofloxacin non-susceptible Escherichia coli and Klebsiella spp. in healthy people in Norway
Source: Antimicrob Resist Infect Control. 2016 Dec 15;5:57. doi: 10.1186/s13756-016-0156-x (PMC5159956; doi:10.1186/s13756-016-0156-x)
Supplement: Additional file 1: — Supplemental material. (DOCX 32 kb) [file 13756_2016_156_MOESM1_ESM.docx]

**Supplemental material**

Table S1: Primers used for detection of ESBL

| Multiplex PCR | Targets | Primer name | Sequence (5’ → 3’) | Amplicon size (bp) | Reference |
| --- | --- | --- | --- | --- | --- |
| ESBL-A | SHV variants including SHV-1 | SHV_for | AGCCGCTTGAGCAAATTAAAC   \|  \|  \| \| --- \| --- \| | 713 | [1] |
|  |  | SHV_rev | ATCCCGCAGATAAATCACCAC |  | [1] |
|  | TEM variants including TEM-1 | TEM_for | GTGCGCGGAACCCCTATT | 822 | [2] |
|  |  | TEM_rev | ACGCTCACCGGCTCCAGATTTAT |  | [3] |
|  | CTX-M variants including CTX-M 15 | CTX-M_for | ATGTGCAGYACCAGTAARGTKATGGC | 593 | [4] |
|  |  | CTX-M_rev | TGGGTRAARTARGTSACCAGAAYCAGCGG |  | [4] |
| AmpC | ACC-1 and ACC-2 | ACC_for | CACCTCCAGCGACTTGTTAC | 346 | [1] |
|  |  | ACC_rev | GTTAGCCAGCATCACGATCC |  | [1] |
|  | FOX-1 to FOX-5 | FOX_for | CTACAGTGCGGGTGGTTT | 162 | [1] |
|  |  | FOX_rev | CTATTTGCGGCCAGGTGA |  | [1] |
|  | MOX-1, MOX-2, CMY-1, CMY-8 to CMY-11 and CMY-19 | MOX_for | GCAACAACGACAATCCATCCT | 895 | [1] |
|  |  | MOX_rev | GGGATAGGCGTAACTCTCCCAA |  | [1] |
|  | DHA-1 and DHA-2 | DHA_for | TGATGGCACAGCAGGATATTC | 997 | [1] |
|  |  | DHA_rev | GCTTTGACTCTTTCGGTATTCG |  | [1] |
|  | LAT-1 to LAT-3, BIL-1, CMY-2 to 7, CMY-12 to CMY-18 and CMY-21 to CMY-23 | CIT_for | CGAAGAGGCAATGACCAGAC | 538 | [1] |
|  |  | CIT_rev | ACGGACAGGGTTAGGATAGY |  | [1] |
|  | ACT-1 and MIR-1 | EBC_for | CGGTAAAGCCGATGTTGCG | 683 | [1] |
|  |  | EBC_rev | AGCCTAACCCCTGATACA |  | [1] |

[1] Dallenne C, Da Costa A, Decre D, Favier C, Arlet G. Development of a set of multiplex PCR assays for the detection of genes encoding important beta-lactamases in Enterobacteriaceae. J Antimicrob Chemother. 2010;65:490-5.

[2] Jones CH, Tuckman M, Keeney D, Ruzin A, Bradford PA. Characterization and sequence analysis of extended-spectrum-{beta}-lactamase-encoding genes from Escherichia coli, Klebsiella pneumoniae, and Proteus mirabilis isolates collected during tigecycline phase 3 clinical trials. Antimicrob Agents Chemother. 2009;53:465-75.

[3] Monstein HJ, Ostholm-Balkhed A, Nilsson MV, Nilsson M, Dornbusch K, Nilsson LE. Multiplex PCR amplification assay for the detection of blaSHV, blaTEM and blaCTX-M genes in Enterobacteriaceae. APMIS. 2007;115:1400-8.

[4] Boyd DA, Tyler S, Christianson S, McGeer A, Muller MP, Willey BM, et al. Complete nucleotide sequence of a 92-kilobase plasmid harboring the CTX-M-15 extended-spectrum beta-lactamase involved in an outbreak in long-term-care facilities in Toronto, Canada. Antimicrob Agents Chemother. 2004;48:3758-64.

Table S2. Frequencies of resistance to antibiotics in ESBL-producing *E. coli* and *Klebsiella* spp. isolated from healthy Norwegians.

| Antibiotic | *E. coli*  n=15 | | |  | *Klebsiella* spp.  n= 1 | | |
| --- | --- | --- | --- | --- | --- | --- | --- |
|  |  |  |  |  |  |  |  |
|  | % R | % I | % S |  | % R | % I | % S |
| Amocixillin-clavulanate | 6.7 | 0 | 93.3 |  | 0 | 0 | 100 |
| Ampicillin* | 100 | 0 | 0 |  | 100 | 0 | 0 |
| Aztreonam | 53.3 | 40 | 6.7 |  | 100 | 0 | 0 |
| Cefotaxime | 93.3 | 6.7 | 0 |  | 100 | 0 | 0 |
| Cefoxitin | 0 | 0 | 100 |  | 0 | 0 | 100 |
| Cefuroxime | 93.3 | 0 | 6.7 |  | 0 | 0 | 100 |
| Ceftazidime | 40 | 33.3 | 26.7 |  | 100 | 0 | 0 |
| Ciprofloxacin | 26.7 | 6.7 | 66.7 |  | 0 | 0 | 100 |
| Gentamicin | 20 | 0 | 80 |  | 0 | 0 | 100 |
| Imipenem | 0 | 0 | 100 |  | 0 | 0 | 100 |
| Mecillinam | 0 | 0 | 100 |  | 0 | 0 | 100 |
| Meropenem | 0 | 0 | 100 |  | 0 | 0 | 100 |
| Nalidixic acid | 80 | 0 | 20 |  | 100 | 0 | 0 |
| Nitrofurantoin | 0 | 0 | 100 |  | 0 | 0 | 100 |
| Piperacillin/tazobactam | 6.7 | 0 | 93.3 |  | 0 | 0 | 100 |
| Trimethoprim | 53.3 | 0 | 46.7 |  | 0 | 0 | 100 |
| Trimethoprim/sulfamethoxazole | 53.3 | 0 | 46.7 |  | 0 | 0 | 100 |

* *Klebsiella* spp. are considered intrinsically resistant to ampicillin.

Table S3. Frequencies of resistance to antibiotics in AmpC -producing *E. coli* isolated from healthy Norwegians.

| Antibiotic | *E. coli* | | |
| --- | --- | --- | --- |
|  | n = 9 | | |
|  | % R | % I | % S |
| Amocixillin-clavulanate | 55.6 | 0 | 44.4 |
| Ampicillin | 55.6 | 0 | 44.4 |
| Aztreonam | 0 | 0 | 100 |
| Cefotaxime | 11,1 | 0 | 88.9 |
| Cefoxitin | 44.4 | 0 | 55.6 |
| Cefuroxime | 33.3 | 0 | 66.7 |
| Ceftazidime | 22.2 | 0 | 77.8 |
| Ciprofloxacin | 33.3 | 0 | 66.7 |
| Gentamicin | 0 | 0 | 100 |
| Imipenem | 0 | 0 | 100 |
| Mecillinam | 0 | 0 | 100 |
| Meropenem | 0 | 0 | 100 |
| Nalidixic acid | 44.4 | 0 | 55.6 |
| Nitrofurantoin | 0 | 0 | 100 |
| Piperacillin/tazobactam | 0 | 0 | 100 |
| Trimethoprim | 0 | 0 | 100 |
| Trimethoprim/sulfamethoxazole | 0 | 0 | 100 |

Table S4. Frequencies of resistance to antibiotics in non-ESBL/AmpC -producing ciprofloxacin non-susceptible *E. coli* and *Klebsiella* spp. isolated from healthy Norwegians.

| Antibiotic | *E. coli* | | |  | *Klebsiella* spp. | | |
| --- | --- | --- | --- | --- | --- | --- | --- |
|  | n= 20 | | |  | n= 4 | | |
|  | % R | % I | % S |  | % R | % I | % S |
| Amocixillin-clavulanate | 40 | 0 | 60 |  | 0 | 0 | 100 |
| Ampicillin* | 75 | 0 | 25 |  | 100 | 0 | 0 |
| Aztreonam | 0 | 0 | 100 |  | 0 | 0 | 100 |
| Cefotaxime | 0 | 0 | 100 |  | 0 | 25 | 75 |
| Cefoxitin | 10 | 0 | 90 |  | 100 | 0 | 0 |
| Cefuroxime | 10 | 0 | 90 |  | 75 | 0 | 25 |
| Ceftazidime | 0 | 0 | 100 |  | 0 | 25 | 75 |
| Ciprofloxacin | 60 | 40 | 0 |  | 0 | 100 | 0 |
| Gentamicin | 20 | 0 | 80 |  | 0 | 0 | 100 |
| Imipenem | 0 | 0 | 100 |  | 0 | 0 | 100 |
| Mecillinam | 10 | 0 | 90 |  | 0 | 0 | 100 |
| Meropenem | 0 | 5 | 95 |  | 0 | 25 | 75 |
| Nalidixic acid | 100 | 0 | 0 |  | 100 | 0 | 0 |
| Nitrofurantoin | 0 | 0 | 100 |  | 100 | 0 | 0 |
| Piperacillin/tazobactam | 5 | 0 | 95 |  | 0 | 75 | 25 |
| Trimethoprim | 25 | 0 | 75 |  | 100 | 0 | 0 |
| Trimethoprim/sulfamethoxazole | 25 | 0 | 75 |  | 75 | 25 | 0 |

* *Klebsiella* spp. are considered intrinsically resistant to ampicillin.

Figure S1. Travel information for visitors to multiple regions during the last 3 (left) and 12 (right) months, from whom resistant isolates were obtained. Each color refer to a WHO region: Africa (orange), America (red), Eastern Mediterranean (green), Europe (outside Scandinavia; blue), South East Asia (purple) and Western Pacific (teal). The number of visitors to each combination of regions is given on the x-axis, together with information about carriage of resistant bacteria: EAP = ESBL or AmpC producing, CNS = ciprofloxacin non-susceptible. Carriers of multiple isolates are represented with one column per isolate per time period, and columns representing the same volunteer are labelled with identical symbols.
